# 3D-printed guiding templates for improved osteosarcoma resection

**DOI:** 10.1038/srep23335

**Published:** 2016-03-21

**Authors:** Limin Ma, Ye Zhou, Ye Zhu, Zefeng Lin, Yingjun Wang, Yu Zhang, Hong Xia, Chuanbin Mao

**Affiliations:** 1 School of Materials Science and Engineering, South China University of Technology, Guangzhou 510641, China; 2 Department of Medical Research, Guangzhou General Hospital of Guangzhou Military Command, 111 Liuhua Road, Guangzhou 510010, China; 3 Department of Orthopedics, Guangdong Key Lab of Orthopaedic Technology and Implant, Guangzhou General Hospital of Guangzhou Military Command, 111 Liuhua Road, Guangzhou 510010, China; 4 Department of Chemistry and Biochemistry, Stephenson Life Sciences Research Center, University of Oklahoma, Norman, OK 73019, USA; 5 School of Materials Science and Engineering, Zhejiang University, Hangzhou, Zhejiang 310027, China

## Abstract

Osteosarcoma resection is challenging due to the variable location of tumors and their proximity with surrounding tissues. It also carries a high risk of postoperative complications. To overcome the challenge in precise osteosarcoma resection, computer-aided design (CAD) was used to design patient-specific guiding templates for osteosarcoma resection on the basis of the computer tomography (CT) scan and magnetic resonance imaging (MRI) of the osteosarcoma of human patients. Then 3D printing technique was used to fabricate the guiding templates. The guiding templates were used to guide the osteosarcoma surgery, leading to more precise resection of the tumorous bone and the implantation of the bone implants, less blood loss, shorter operation time and reduced radiation exposure during the operation. Follow-up studies show that the patients recovered well to reach a mean Musculoskeletal Tumor Society score of 27.125.

Over the past 5 years, computer-assisted design (CAD) of guiding templates has been used effectively for the placement of lumbar pedicle, C2 laminar, cervical pedicle, and thoracic pedicle screws, as well as in iliosacral screw placement and the correction of orthopedic cubitus varus deformity^1^,^2^,^3^,^4^,^5^,^6^. Computed tomography (CT) and magnetic resonance imaging (MRI) are essential preoperative studies before complex osteosarcoma surgery, as they are needed to determine the anatomical structures for surgical planning. However, recognition of the actual extent of osteosarcoma margins using preoperative CT and/or MRI has proved very difficult, and inaccurate surgical resection of the lesion might cause local recurrence and distant metastasis^7^,^8^. Hence, resection of such tumors has turned out to be challenging. Surgery planning is recommended to correctly estimate the size of the tumor and development of an allograft-prosthetic composite that precisely fits the area of resection so as to ensure good anchoring during osteosarcoma resection. Recently, CAD-rapid prototyping (CAD-RP) surgery for malignant tumors has been developed to facilitate precise resection and accurate reconstruction, and has yielded satisfactory results^9^. Attempts to use surgical guides have recently been reported in the orthopedic fields^8^,^10^,^11^,^12^. However, until now, there is no clinical report on the efficacy of guiding templates in high-grade osteosarcoma resection and the recovery of the patients following the resection.

Therefore, the objectives of this work are to use CAD to design guiding templates for osteosarcoma, to use 3D printing technique to fabricate the guiding templates, and to evaluate the use of the guiding templates in the clinical surgery of high-grade osteosarcoma with high accuracy. The scientific question we want to address is whether the use of the 3D-printed guiding templates will enable not only more precise resection of the tumorous bone and the implantation of the bone implants, less blood loss, shorter operation time and reduced radiation exposure during the operation, but also the good recovery of the patients.

## Materials and Methods

### Three-dimensional (3D) reconstruction of the bone model

**Figure 1:**
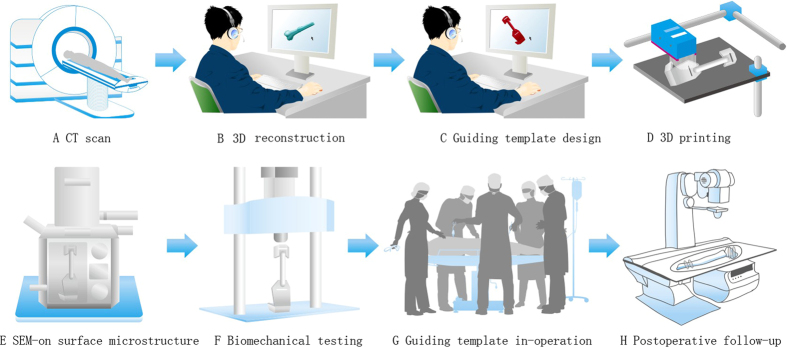
Schematic illustration of the general idea of this study. CT scan is first performed on the tumorous bone (**A**). The CT scan image is then used to reconstruct a 3D model of the tumorous bone using Simpleware software (**B**). The 3D model is then used to design a guiding template using reverse engineering software (**C**). The model of the guiding template is then used to fabricate the template by 3D printing (**D**). The 3D printed template is characterized by SEM imaging (**E**) and biomechanical testing (**F**). The template is further used to guide the surgery of osteosarcoma (**G**). The patient is followed after surgery to test the efficacy of the surgery (**H**).

**Figure 2:**
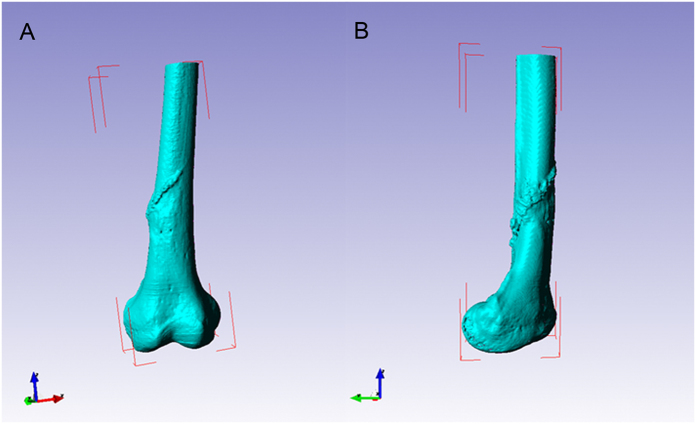
Three-dimensional reconstruction of osteosarcoma bone models using Scan IP software. (**A**) Frontal view. (**B**) Lateral view.

A Siemens dual-source 64-slice spiral CT (SOMATOM Definition CT, Siemens Healthcare, Forchheim, Germany) (Fig. 1A) was used for continuous axial tomography of the full knee joint with 0.625-mm slice thickness and 0.35-mm in-plane resolution, with scanning parameters set at 120 kV and 205.50 mAs. The scanning matrix size was 512 × 512. The knee joint region was continuously scanned to reach 266 layers. Images were processed into data files in Digital Imaging and Communications in Medicine (DICOM) format, and transferred to a workstation running Simpleware 6.0 software (Simpleware Ltd, Exeter, United Kingdom) to generate a 3D reconstruction model for the desired femur bone model (Figs 1B and 2).

**Figure 3:**
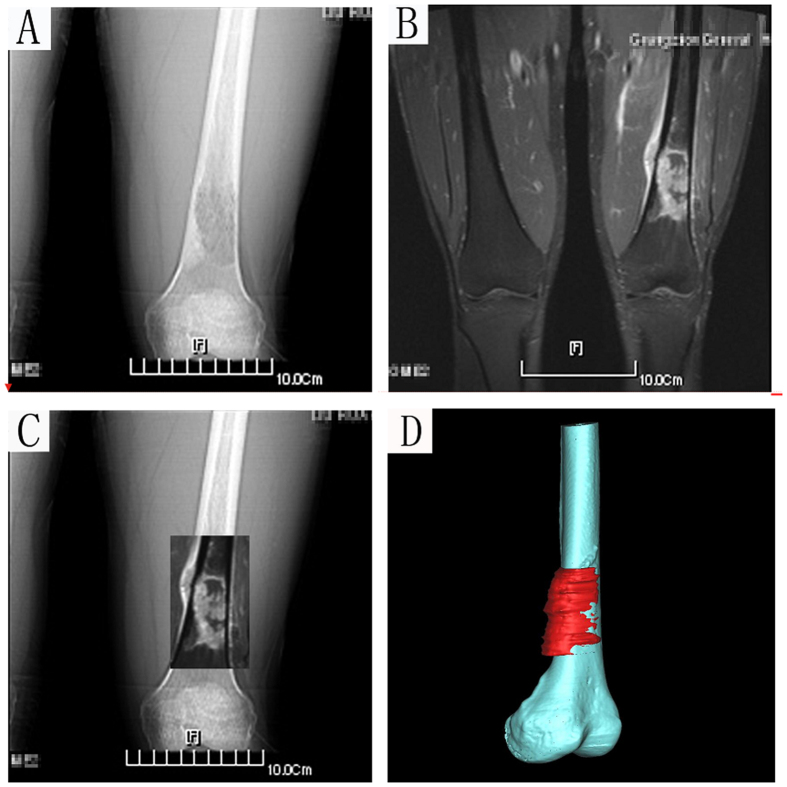
A 21-year-old patient with osteosarcoma of the distal femur. (**A**) Anteroposterior CT scan image. (**B**) MRI image. Both CT and MRI images revealed an osteosarcoma in the left distal femur. (**C**) Merging of the CT and MRI images for the delineation of the tumor. (**D**) The 3-D bone tumor model reconstructed from the merged CT and MRI image with the tumor profile highlighted in red.

Preoperative CT and MRI examinations of each patient were performed. One week prior to surgery, CT and MRI examinations were carried out to obtain two-dimensional (2D) CT (Fig. 3A) and MRI data of the lesion (Fig. 3B), respectively. MRI images of the corresponding region were acquired using a 1.5-T unit (Siemens Sonata; Siemens Medical Solutions, Erlangen, Germany). Postcontrast T1-weighted axial images (TR, 512 milliseconds; TE, 13 milliseconds; 2-mmthick slices) were merged with CT images for better bone-soft tissue contrast. The merging of the CT and MRI images (Fig. 3C) guided the preoperative simulation processes including CAD design of the template assisted tumor resection and 3D reconstruction of the femur with the tumor profile highlighted in red (Fig. 3D).

### Manufacture of individual guiding templates using 3D printing

**Figure 4:**
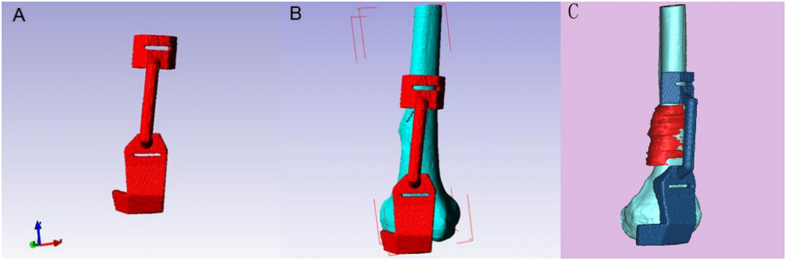
Design of the guiding template on the basis of the 3D tumorous bone shown in Fig. 1 using reverse engineering software according to anatomical position. (**A**) The three-dimensional computer model of the guiding template. (**B**) The guiding template fitted the distal femur perfectly. (**C**) Target resection planes were designed according to the osteosarcoma (red) with a safe margin.

**Figure 5:**
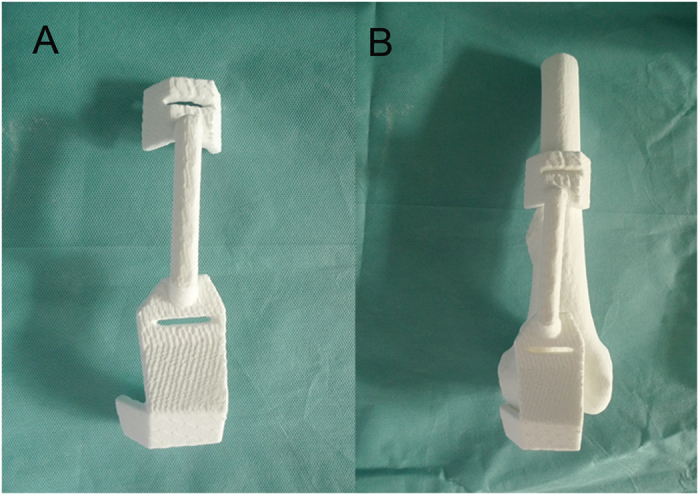
3D printed guiding template (**A**) and the combination of the template and femur (**B**) shows that the guiding template fitted the rapid prototyping model of the distal femur perfectly.

The 3D femur model was exported in stereolithographic (STL) format and opened in a workstation running Reverse Engineering (RE) software UG image-ware 12.0 (EDS Co., USA) in order to determine the optimal position for tumor resection. The resection margin was extended 3–5 cm beyond the perimeter of the actual tumors in bone tissue. The design of the guiding template (Figs 1C and 4) was based on the findings of previous studies^2^,^8^. The guiding template was fabricated by 3D printing of the commercial MED610 purchased from Objet Ltd, Israel (Figs 1D and 5). The guiding template used for the three-dimensional (3D) test had an incision (25 mm long, 6 mm thick, and 5 mm wide).

### Characterization of the property of the templates

#### Scanning electron microscopy

The surface morphology of the samples (1 cm in diameter and 1 mm in thickness) was examined by field-emission scanning electron microscopy (SEM, Zeiss Evols 10, Germany) at an accelerating voltage of 15 kV (Fig. 1E).

#### Determination of compressive properties

A mechanical testing machine (Instron5967, INSTRON, USA) was used to test samples. Prior to each test, the samples were fabricated into a cylinder about 1 cm in diameter and 2 cm in height. The loading speed was set as 1 mm/min (Fig. 1F). When the load pressure declined, the compression stopped and the maximum compression pressure was determined by the peak value of compressive curve where the pressure reduction was more than 5%.

#### Cytocompatibility

We followed our published protocol to evaluate the cytocompatibility of the guiding templates^13^,^14^. Specifically, MC3T3-E1 (ATCC) cells were cultured on a small piece of the guiding templates in α-modified Eagle’s medium (α-MEM) supplemented with 10% fetal bovine serum (FBS) in a humidified atmosphere of 5% CO_2_ at 37 °C. Cell proliferation was tested by cell counting kit-8 (CCK-8, Dojindo, Japan). At the indicated time points (1d, 3d, 5d), 300 μl of 10% CCK-8 solution was added to the plates and incubated for 2 h at 37 °C. The optical density was read on an ELISA plate reader at 450 nm. The experiments where cells were not cultured on the guiding templates were used as control.

### Surgery using 3D printed guiding templates

#### Research ethics approval

This study was conducted in accordance with the principles outlined in the Declaration of Helsinki and was approved by the Ethics Committee of Guangzhou General Hospital of Guangzhou Military Command. Written informed consent was obtained from all patients involved in the study (or from their parents or guardians if they were aged less than 18 years). Patient data were kept anonymous to ensure confidentiality and privacy.

### Patient characteristics

Between September 2011 and November 2013, 8 patients (5 male and 3 female, aged 10–25 years) with osteosarcoma pathology requiring resection underwent posterior surgery of the knee joint. Patient demographics and mean follow-up times are listed in Tables 1 and 2.

#### Surgery

**Figure 6:**
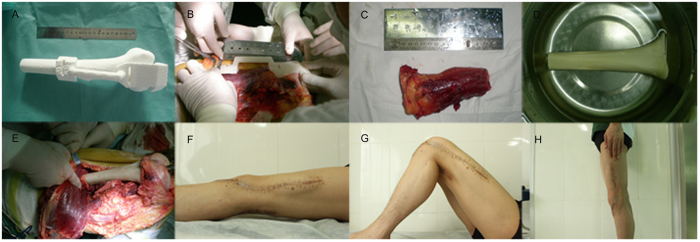
The guiding template applied in the operation on an 18-year-old man with a distal femur. (**A**) 3D printed guiding template fitted with the femur model. (**B**) The guiding template fitted perfectly with the distal femur during the operation and provided anatomical guidance for a safe osteotomy line. (**C**) Precise excision of the tumorous bone according to the surgery guided by the template. (**D**) The pruned allografted bone to be implanted. (**E**) The allograft was implanted into the bone defect. (**F–H**) The patient was healed (**F**, Supine view; **G**, Flexion view; **H**, Standing view).

After induction of general anesthesia, the surgical area was prepared and draped, and the tumor was sufficiently exposed. After appropriate preparation, the 90° flexion of the left knee was located. During the surgery, the sterile model and guiding template were used to determine the range of the osteotomy. The tumor was surgically removed with the use of guiding templates. Using the template, a large allogeneic bone obtained before surgery was trimmed into a 3D shape matching the bone defect after tumor resection. The guiding template was used to perform distal transverse osteotomy and prune the allografted bone (Figs 1G and 6). The allograft was implanted into the bone defect and fixed with an intramedullary nail and 4 lock nails. In each case, the duration of surgery and intraoperative blood loss were recorded.

#### Postoperative rehabilitation

In each case, ankle extension and flexion exercises were started on postoperative day 1, hip flexion and knee flexion exercises were begun on postoperative day 3, and straight leg raises were conducted on postoperative day 7. The patient was encouraged to walk with crutches 10 days after surgery. The patient was able to walk independently 3 months after surgery.

## Results

### Characterization of 3D guiding template

As auxiliary medical devices, the guiding templates will be in contact with the human body such as the skin when being used clinically. Therefore, it is better for the templates to have good biocompatibility. The template should also bear good mechanical property because surgeons exert vertical force on the templates during operation. Hence, the templates need to have good strength and tenacity to avoid fracture. Therefore, we characterized the mechanical properties and biocompatibility.

**Figure 7:**
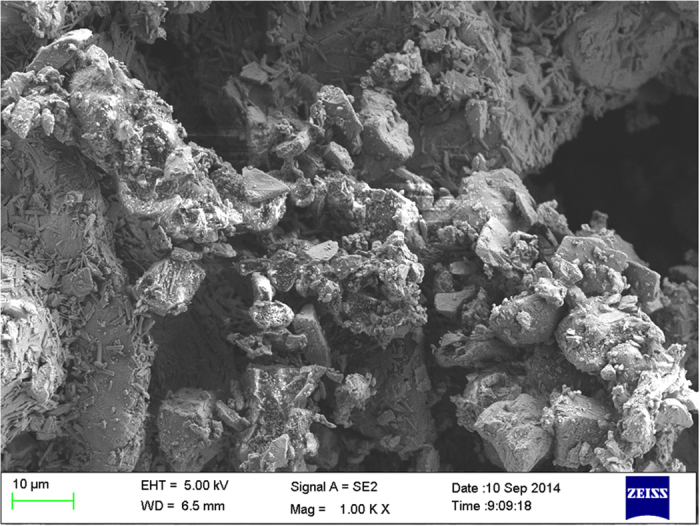
Surface morphology of the 3D guiding template surface.

**Figure 8:**
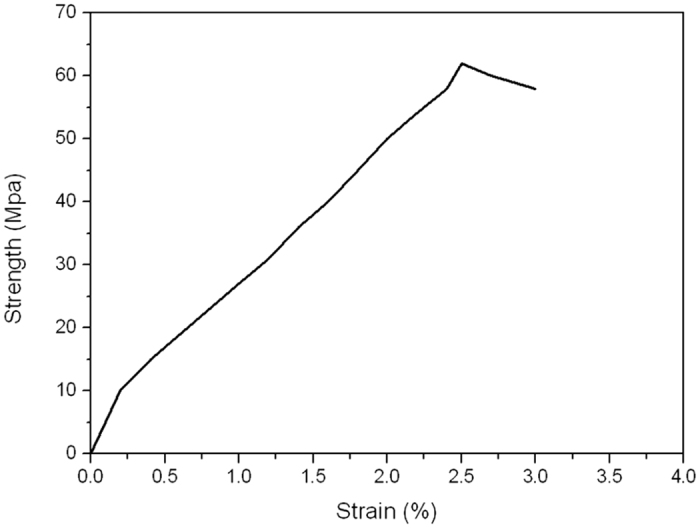
The stress-strain curve of the template.

Figure 7 showed the surface morphology of the 3D guiding template, which indicated that a compact scaffold had been successfully fabricated. The mechanical properties of the guiding template were further characterized. As can be seen from the results (Fig. 8), the maximal compressive strength of the template (60.01 ± 2.52 MPa).

**Figure 9:**
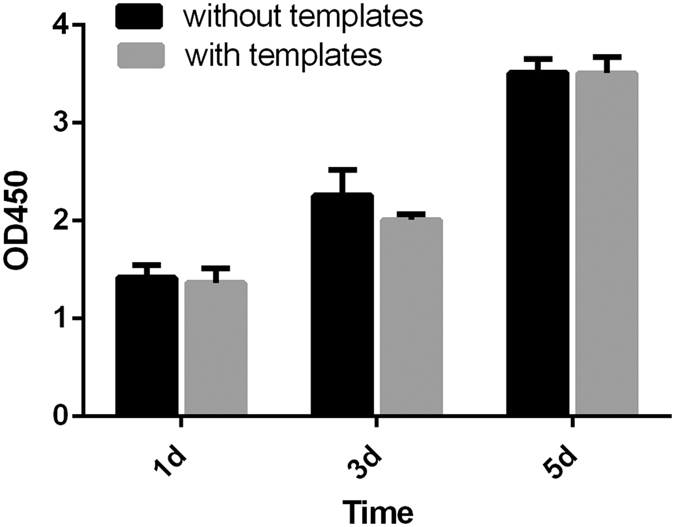
Cell proliferation on the guiding templates after 1, 3 and 5 days of incubation was measured by colorimetric CCK-8 assay. The group cultured in the absence of the guiding templates but with α-MEM supplemented with 10% FBS serving as a control. The results shown are the means ± standard deviation of three separate experiments performed in triplicate.

Cell proliferation during the first 5 days of incubation with the guiding templates was assessed as showed in Fig. 9. On day 1, cells cultured in the presence or absence of the guiding plates showed a relatively lower absorbance in comparison with those at later time points. After 3 days, the absorbance measurements of all groups increased drastically, indicating that the number of living cells was increased. However, during the first 5 days, the cell numbers in the presence of the guiding plates showed no statistically significant differences compared with the control group (p > 0.05) (Fig. 9).

### Clinical study

In all cases, the optimal position for resection was determined by the corresponding 3D printed guiding template. Each guiding template fitted its corresponding femur model perfectly. The entry point and direction for the osteosarcoma resection were therefore chosen based on the guiding templates, providing accuracy and convenience during the procedure. In conventional surgery without the use of the guiding template^15^, the average operation time is 217 min (300-420 min in our hospital), the average volume of blood loss is 1025 ml (1000-2000 ml in our hospital), and the size of the surgical incision in our hospital varies between 250 and 350 mm. In contrast, in the surgery guided by the 3D printed guiding template, the operation time, blood loss and incision size ranged 180–250 min, 560–900 ml, and 115–180 mm, respectively (Table 1).

**Figure 10:**
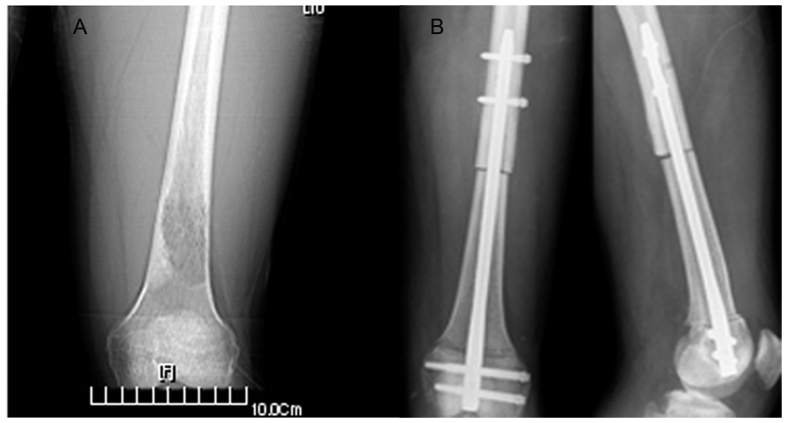
X-ray image showing that the interlocking intramedullary nails were excellent, with high stability. (**A**) Preoperative and (**B**) postoperative radiographs (Left, front view; right, lateral view).

All patients were followed up from 25 to 42 months (mean, 33 months), and all 8 patients were alive at the time of reporting. The Musculoskeletal Tumor Society (MSTS) score was between 21 and 30 at the last follow-up^16^, and the mean knee flexion was 112.5° (range, 90°–130°, Table 2). It should be noted that the MSTS score is a common method for evaluating the postoperative evaluation of osteosarcoma surgery^16^. At the 2-year follow-up check, the patient was alive and living well without the evidence of recurrence. A plain X-ray film revealed that the bone defect was healed and no bone tumor was identified (Figs 1H and 10). The X-ray scan showed that using the individual templates resulted in the surgery with a high degree of precision, but without the cases of intramedullary nail misplacement (Fig. 10). The implants were stabilized and could bear sufficient weight to allow patients to eventually recover normal physical activity.

## Discussion

Guiding templates have been applied in the field of orthopaedic oncology in recent years. It may facilitate resection and reconstruction in patients with complex bone tumor^8^,^11^,^12^. Inadequate resection margins are associated with higher risk of local tumor recurrence and poorer patient survival^17^. For example, Jeys *et al.* recently reported the largest series of 31 cases with pelvic or sacral tumors operated with the computer-assisted tumor surgery technique, but the local recurrence rate was 13% at a mean follow-up of 13.1 months^18^. In other studies on the computer-assisted tumor surgery^19^,^20^, the local recurrence rates were reported to be 20–25% at a minimum of 3 years of follow-up.

In order to achieve safe and accurate tumor resections, it is necessary to establish the profile of the tumor in the bone and soft tissues. Due to the complexity associated with the musculoskeletal bone tumor resections and reconstructions, tumor surgeons usually used 2D CT and MRI images for surgical planning. Such planning is difficult without the help of a computer due to the more complex regional anatomy in osteosarcoma. In recent years, surgery using navigation systems has helped to facilitate resection and reconstruction in patients with complex osteosarcoma, and several preliminary reports have described its application in bone tumor surgery^17^,^20^,^21^,^22^,^23^,^24^. These reports involve the resection of pelvic and sacral tumors and joint preserving limb salvage surgery^20^,^23^,^24^. The surgical navigation systems greatly improved the accuracy and safety of implantation and reduced neural, visceral, and vascular complications. Nevertheless, these systems still have a number of disadvantages including increased cost, extended surgical time, additional radiation exposure, cumbersome surgical procedures, and significant learning curves. Registration-related errors and changes in the patient’s position can also have a negative impact on the accuracy of surgical navigation systems.

In this study, we used 3D printing technique to manufacture the guiding templates for high grade osteosarcoma. We used a combination of CT scan and MRI imaging to obtain the model of the tumors and then employed the model to produce the guiding templates by 3D printing technique. The cell proliferation studies clearly demonstrated that the guiding templates are biocompatible. The characterization of the mechanical properties also showed that the guiding templates have good compressive strength to sustain the surgery process. It was reported that using surgical guides could improve the accuracy of resection of malignant bone tumors^8^,^11^. It was also suggested that the osteotomy guides achieved by novel 3D printing technologies could reduce the treatment costs and production time^12^. Compared to these studies, our study not only characterized the biological and materials properties of the guiding templates but also resulted in shorter operation time, less blood loss and smaller incision size.

We then used 3D printed guiding templates to assist the surgery of osteosarcoma and yielded satisfactory results. Specifically, the use of the 3D guiding templates has led to precise resection of the bone and implantation of allografts, less blood loss, shorter surgery time and reduced radiation (Tables 1 and 2). In addition, it took about 10 h to manufacture a 3D printed guiding template and femur model, and the cost of such manufacture was estimated to be about $30. Namely, the use of the guiding templates is cost-efficient. Our study shows that the patients subjected to the surgery guided by the guiding templates recovered in 3 months.

Although our studies show the advantages of the guiding templates, there are still some limitations of our study. First, the primary limitations of this study are the small number of cases and the relatively short follow-up time. Second, the potential benefits of the surgery guided technique in improving surgical accuracy may help reduce the risk of local recurrence but may not translate into better patient survival for metastatic disease. Third, the control study without using the guiding templates could not be performed on the same patients.

## Conclusion

In conclusion, personalized guiding templates for osteosarcoma resection provide surgeons with a novel tool for optimizing allograft bone construction and thus minimizing overall tissue trauma and reducing the risk of damaging nervous and vascular structures. The guiding templates, manufactured by 3D printing technique, are biocompatible and have good compressive strength. The current study demonstrates that the application of such guiding templates can result in shorter surgical duration, lower radiation exposure and less blood loss. It also makes the surgery easier to perform. With its wide applicability, high accuracy, and cost-effectiveness, the use of this technology is expected to become widespread in the future.

## Additional Information

**How to cite this article**: Ma, L. *et al.* 3D-printed guiding templates for improved osteosarcoma resection. *Sci. Rep.* **6**, 23335; doi: 10.1038/srep23335 (2016).

## Figures and Tables

**Table 1:** Demographic data of eight cases of surgical trauma.

Patient number	Age (years)/sex	Operation Time (minute)	Blood loss (ml)	Length of incision (mm)
1	12/F	180	560	115
2	14/M	200	760	130
3	17/M	240	660	160
4	18/M	260	640	150
5	21/M	160	840	180
6	23/M	210	900	170
7	25/F	200	820	175
8	10/F	250	790	155

**Table 2:** Details of eight cases operated with computer-assisted guiding template.

Patient number	Age (years)/sex	Location	ROM (°)	MSTS score (points)	Follow-up (months)
1	12/F	Distal Femur	0–105	27	25
2	14/M	Distal Femur	0–90	21	42
3	17/M	Distal Femur	0–120	29	36
4	18/M	Distal Femur	0–130	30	30
5	21/M	Distal Femur	0–100	26	35
6	23/M	Distal Femur	0–110	28	26
7	25/F	Distal Femur	0–105	27	38
8	10/F	Distal Femur	0–125	29	32

^*^Total score is 30; MSTS = Musculoskeletal Tumor Society score; M = male; F = female.

## References

[b1] Lu S. *et al.* A novel patient-specific navigational template for cervical pedicle screw placement. Spine. 34, E959–966 (2009). 20010385 10.1097/BRS.0b013e3181c09985

[b2] Lu S. *et al.* A novel computer-assisted drill guide template for lumbar pedicle screw placement: a cadaveric and clinical study. Int J Med Robot. 5, 184–191 (2009). 19280584 10.1002/rcs.249

[b3] Lu S. *et al.* A novel computer-assisted drill guide template for placement of C2 laminar screws. Eur Spine J 18, 1379–1385 (2009). 19517142 10.1007/s00586-009-1051-4 PMC2899526

[b4] Zhang Y. Z. *et al.* Application of computer-aided design osteotomy template for treatment of cubitus varus deformity in teenagers: a pilot study. J Shoulder Elbow Surg. 20, 51–56 (2011). 21134665 10.1016/j.jse.2010.08.029

[b5] Ma T. *et al.* A novel computer-assisted drill guide template for thoracic pedicle screw placement: a cadaveric study. Arch Orthop Trauma Surg. 132, 65–72 (2012). 21874371 10.1007/s00402-011-1383-5

[b6] Chen B., Zhang Y., Xiao S., Gu P. & Lin X. Personalized image-based templates for iliosacral screw insertions: a pilot study. Int J Med Robot. 8, 476–482 (2012). 22893233 10.1002/rcs.1453

[b7] Schwab J. H., Healey J. H., Rose P., Casas-Ganem J. & Boland P. J. The surgical management of sacral chordomas. Spine. 34, 2700–2704 (2009). 19910774 10.1097/BRS.0b013e3181bad11d

[b8] Bellanova L., Paul L. & Docquier P. L. Surgical guides (patient-specific instruments) for pediatric tibial bone sarcoma resection and allograft reconstruction. Sarcoma. 2013, 787653 (2013). 23533326 10.1155/2013/787653 PMC3603296

[b9] Zhang Y. *et al.* Surgical treatment of pelvic malignant tumors with individualized hemi-pelvic. Orthop. J. China. 17, 190–192 (2009).

[b10] Wong K. C., Kumta S. M., Sze K. Y. & Wong C. M. Use of a patient-specific CAD/CAM surgical jig in extremity bone tumor resection and custom prosthetic reconstruction. Comput Aided Surg. 17, 284–293 (2012). 23030839 10.3109/10929088.2012.725771

[b11] Gouin F. & Paul L. Computer-Assisted Planning and Patient-Specific Instruments for Bone Tumor Resection within the Pelvis: A Series of 11 Patients. Sarcoma. 2014, 842709 (2014). 25100921 10.1155/2014/842709 PMC4101950

[b12] Holzapfel B. M. *et al.* Customised osteotomy guides and endoprosthetic reconstruction for periacetabular tumours. Int Orthop. 38, 1435–1442 (2014). 24658873 10.1007/s00264-014-2314-1 PMC4071499

[b13] Yang M. *et al.* Biomimetic nucleation of hydroxyapatite crystals mediated by Antheraea pernyi silk sericin promotes osteogenic differentiation of human bone marrow derived mesenchymal stem cells. Biomacromolecules. 15, 1185–1193 (2014). 24666022 10.1021/bm401740x PMC3993896

[b14] Hao L. *et al.* Directing the fate of human and mouse mesenchymal stem cells by hydroxyl-methyl mixed self-assembled monolayers with varying wettability. J Mater Chem B Mater Biol Med. 2, 4794–4801 (2014). 25328680 10.1039/C4TB00597J PMC4196441

[b15] Saidi K. *et al.* Supracondylar periprosthetic fractures of the knee in the elderly patients: a comparison of treatment using allograft-implant composites, standard revision components, distal femoral replacement prosthesis. J Arthroplasty. 29, 110–114 (2014). 23680503 10.1016/j.arth.2013.04.012

[b16] Wong K. C., Kumta S. M., Antonio G. E. & Tse L. F. Image fusion for computer-assisted bone tumor surgery. Clin Orthop Relat Res. 466, 2533–2541 (2008). 18648902 10.1007/s11999-008-0374-5 PMC2584299

[b17] Picci P. *et al.* Relationship of chemotherapy-induced necrosis and surgical margins to local recurrence in osteosarcoma. J Clin Oncol. 12, 2699–2705 (1994). 7989947 10.1200/JCO.1994.12.12.2699

[b18] Jeys L., Matharu G. S., Nandra R. S. & Grimer R. J. Can computer navigation-assisted surgery reduce the risk of an intralesional margin and reduce the rate of local recurrence in patients with a tumour of the pelvis or sacrum? Bone Joint J. 95-b, 1417–1424 (2013). 24078543 10.1302/0301-620X.95B10.31734

[b19] Cho H. S., Oh J. H., Han I. & Kim H. S. The outcomes of nvigation-assisted bone tumour surgery: minimum three-year follow-up. J Bone Joint Surg Br. 94, 1414–1420 (2012). 23015571 10.1302/0301-620X.94B10.28638

[b20] Wong K. C. & Kumta S. M. Computer-assisted tumor surgery in malignant bone tumors. Clin Orthop Relat Res. 471, 750–761 (2013). 22948530 10.1007/s11999-012-2557-3 PMC3563803

[b21] Wong K. C. *et al.* Precision tumour resection and reconstruction using image-guided computer navigation. J Bone Joint Surg Br. 89, 943–947 (2007). 17673591 10.1302/0301-620X.89B7.19067

[b22] Cho H. S., Kang H. G., Kim H. S. & Han I. Computer-assisted sacral tumor resection. A case report. J Bone Joint Surg Am. 90, 1561–1566 (2008). 18594106 10.2106/JBJS.G.00928

[b23] Cho H. S., Oh J. H., Han I. & Kim H. S. Joint-preserving limb salvage surgery under navigation guidance. J Surg Oncol. 100, 227–232 (2009). 19330812 10.1002/jso.21267

[b24] Hufner T. *et al.* New indications for computer-assisted surgery: tumor resection in the pelvis. Clin Orthop Relat Res. 219–225 (2004). 15346077 10.1097/01.blo.0000138958.11939.94

